# Interleukin-6 Expression by Hypothalamic Microglia in Multiple Inflammatory Contexts: A Systematic Review

**DOI:** 10.1155/2019/1365210

**Published:** 2019-08-22

**Authors:** Vanessa C. D. Bobbo, Carlos P. Jara, Natália F. Mendes, Joseane Morari, Lício A. Velloso, Eliana P. Araújo

**Affiliations:** ^1^Faculty of Nursing, University of Campinas, SP 13083-887, Brazil; ^2^Laboratory of Cell Signaling, Obesity and Comorbidities Research Center, University of Campinas, SP 13083-864, Brazil

## Abstract

Interleukin-6 (IL-6) is a unique cytokine that can play both pro- and anti-inflammatory roles depending on the anatomical site and conditions under which it has been induced. Specific neurons of the hypothalamus provide important signals to control food intake and energy expenditure. In individuals with obesity, a microglia-dependent inflammatory response damages the neural circuits responsible for maintaining whole-body energy homeostasis, resulting in a positive energy balance. However, little is known about the role of IL-6 in the regulation of hypothalamic microglia. In this systematic review, we asked what types of conditions and stimuli could modulate microglial IL-6 expression in murine model. We searched the PubMed and Web of Science databases and analyzed 13 articles that evaluated diverse contexts and study models focused on IL-6 expression and microglia activation, including the effects of stress, hypoxia, infection, neonatal overfeeding and nicotine exposure, lipopolysaccharide stimulus, hormones, exercise protocols, and aging. The results presented in this review emphasized the role of “injury-like” stimuli, under which IL-6 acts as a proinflammatory cytokine, concomitant with marked microglial activation, which drive hypothalamic neuroinflammation. Emerging evidence indicates an important correlation of basal IL-6 levels and microglial function with the maintenance of hypothalamic homeostasis. Advances in our understanding of these different contexts will lead to the development of more specific pharmacological approaches for the management of acute and chronic conditions, like obesity and metabolic diseases, without disturbing the homeostatic functions of IL-6 and microglia in the hypothalamus.

## 1. Introduction

The identification of leptin uncovered the important role of the brain in the regulation of whole-body energy homeostasis [1], which has had a tremendous impact on our understanding of the underlying physiopathology of obesity and a number of related metabolic diseases [2–4]. The hypothalamus, in particular, plays an important role in metabolic regulation, and studies have demonstrated its critical role in the regulation of energy balance by integrating peripheral hormone and neuronal signals of satiety and nutritional status, as well as by directly sensing nutrients [5–8].

The leptin-melanocortin pathway is considered the most robust regulator of whole-body energy homeostasis [9, 10]. This function is provided by two counteracting populations of neurons in the arcuate nucleus of the hypothalamus (ARC), the first of which is proopiomelanocortin (POMC), which has an anorexigenic role, and the second is agouti-related peptide (AgRP)/neuropeptide Y (NPY), which has orexigenic action [3, 11]. These neuronal populations are sensitive to afferent inputs, like leptin and insulin, which regulate both acute and long-term energetic states.

Studies of diet-induced obesity (DIO) and aging have shown that the hypothalamus is targeted by an inflammatory process that leads to defective regulation of energy homeostasis [12–15]. By activating signal transduction through toll-like receptor 4 (TLR4), long-chain saturated fatty acids (SFAs) induce an inflammatory response in the hypothalamus [16]. Signaling through JNK and NF-kB not only increases the mRNA levels of TNF-*α*, interleukin- (IL-) 1*β*, IL-6, and IFN-*γ* cytokines, but can also lead to endoplasmic reticulum stress, autophagy, and mitochondrial dysfunction [13, 17, 18].

Among the cytokines identified in the hypothalamus, IL-6 has gained considerable attention in studies related to metabolism due to its pleiotropic actions, not only in the pathogenesis of inflammatory disorders, but also in the physiological homeostasis of nervous tissue [19]. IL-6 can stimulate responses in a given target cell in two different ways. The classical signaling pathway corresponds to the binding of IL-6 to its membrane-bound *α*-receptor, IL-6R, resulting in dimerization of its *β*-receptor gp130. Alternatively, transsignaling is activated when IL-6 binds to the soluble IL-6R fraction, and this complex can then stimulate distant cells that express gp130 but not surface-bound IL-6R [20, 21]. Both pathways lead to the activation of downstream JAK/STAT signaling, which upregulates the transcription of proinflammatory genes [22, 23]. Although IL-6 levels in the brain are low under physiological conditions, its levels have been reported to be increased in several neurological disorders, predominantly due to neuronal and glial cells [24].

Microglial cells are the resident macrophages of the central nervous system (CNS) and are widely distributed throughout the brain. They originated from primitive macrophages in the yolk sac and form a population that is distinct from bone marrow-derived macrophages (BMDM) [25, 26]. Under normal physiological conditions, they are relatively quiescent; however, after being exposed to injury or infection stimuli, they undergo morphological and functional changes [26]. Many mechanisms by which microglia can be activated have been described [27, 28]. In the mediobasal hypothalamus (MBH), glial cells, which also include astrocytes, have been implicated in initiating and propagating an inflammatory process resulting in gliosis [29, 30]. Gliosis is characterized by an increased number of glial cells, hypertrophy of the cell bodies and processes, and other physiological changes. It occurs, in part, because microglial cells are able to sense changes in the surrounding environment and can quickly become activated to either a proinflammatory (M1) or anti-inflammatory (M2) phenotype. When activated, microglia release cytokines, including IL-6, in addition to other chemokines and growth factors, in order to mitigate or prevent damage to the brain due to the insult.

Although there has been significant progress in studies involving glial cells and cytokines, especially in areas of the brain that are important for metabolic physiologic control or neurodegenerative diseases, the relationship between microglia and IL-6 in the brain remains unclear. In this review, we searched for studies that evaluated IL-6 from a microglial origin in the hypothalamic environment. Data from articles were systematically reviewed to identify the hypothalamic microglia status (whether activated and/or the source of IL-6) and IL-6 expression (whether increased, unaltered, or decreased) in multiple conditions related to inflammatory/pathological processes.

*PICOS Strategy*. Participants: murine model. Interventions: conditions related to inflammatory and pathological processes. Comparisons: Hypothalamic microglia status. Outcomes: IL-6 expression. Study design: Experimental studies.

## 2. Materials and Methods

### 2.1. Search Strategy

A systematic search was performed in PubMed and Web of Science databases on December 18, 2018, for published studies on the association between IL-6 and microglia in the hypothalamus, with no restrictions with regard to language, timespan, or document type, using mixed strategy keywords. The Systematic Review Protocol was registered in “International Prospective Register of Systematic Reviews” (PROSPERO) through the code CRD42019129248. The following search strategies were used: PubMed, ((“Hypothalamus”[Mesh]) AND “Interleukin-6”[Mesh]) AND “Microglia”[Mesh]; Web of Science, ALL FIELDS: (“interleukin 6” and “microglia” and “hypothalamus”). This review follows the “Preferred Reporting Items for Systematic Reviews and Meta-Analyses” (PRISMA) checklist (*See PRISMA Checklist*, [Supplementary-material supplementary-material-1]).

After obtaining the search results, duplicate articles, those that exclusively used an* in vitro* approach, studies without data on IL-6 levels, and those without information regarding microglia in the hypothalamus were identified and excluded from this review (*See Table of included and excluded articles,*[Supplementary-material supplementary-material-1]).

### 2.2. Data Extraction and Classification

The following data were extracted from each study: subject of study, model adopted (*in vivo* and* in vitro*) and intervention (treatment or exposure performed), methods of analysis (mRNA expression and protein expression), IL-6 expression and microglia status (increased/decreased or activated/suppressed in comparison to control) with tissue/location, expression of IL-6 by microglia, and phenotypic outcome. When available, the significance level (p-value) was collected. The evaluation of risk of bias was performed using the SYRCLE's risk of bias tool (*See Evaluation of Risk of Bias, using SYRCLE's risk of bias tool,*[Supplementary-material supplementary-material-1]), developed for animal studies.

## 3. Results

The search strategy identified a total of 25 articles (PubMed, n = 13; Web of Science, n = 13, of which 12 were unique). Twelve papers were excluded based on their title and abstract. The remaining 13 studies were retrieved for a full evaluation and were confirmed to fulfill the inclusion criteria (*See Flow diagram of Systematic Review provided by PRISMA*, [Supplementary-material supplementary-material-1]). Details of these 13 studies are summarized in Table 1.

### 3.1. Increased Hypothalamic IL-6 Expression and Microglial Status

In this review, the publication search returned eight articles that reported increased IL-6 expression. In five of these articles, increased hypothalamic levels of IL-6 with microglia activation were described. These events were found after (a) exposure to amylin, which is synthesized by pancreatic *β*-cells and is coreleased with insulin in response to food intake and increased glucose concentrations [31]; (b) lung coinfection, which induces neuroinflammatory events, in part through serum amyloid A production [32]; (c) lipopolysaccharide (LPS) exposure in neonatally overfed adults [33] and in glial cells from P2X7R-knockout mice (purinoceptor expressed predominantly by cells with immune origin) [34]; and (d) neonatal overfeeding itself [35].

One study showed an increase in hypothalamic IL-6 expression in the context of microglial suppression. Induced hypoxia leads to an increase in IL-6 levels, even with suppression of microglial activity in the CNS (pharmacological suppression). Thus, the increased IL-6 expression was probably from other cell types, like astrocytes. Altered microglial activation was related to alterations in the brain autonomic nuclei responsible for cardiorespiratory control, leading to impairments in breathing [36].

Finally, three studies showed increased hypothalamic IL-6 expression but did not measure microglial activation. Similar to amylin [31], another hormone known as mimecan (also known as osteoglycin) can lead to an increase in hypothalamic IL-6 expression. Using* in vitro* approaches, this study showed that IL-6 is produced by microglia after mimecan stimulus. Mimecan is expressed in adipose tissue, and its action is related to the inhibition of food intake and reduction of body weight in mice [37]. The final study, which employed a model of early-life stress, found increased hypothalamic IL-6 expression [38].

### 3.2. Unaltered or Decreased Hypothalamic IL-6 Expression and Microglial Status

The expression of IL-6 was unaltered in three studies related to (a) aging as a physiological condition, which leads to an increase in IL-6 levels in other brain areas, but no significant increase was observed in the hypothalamus [39]; (b) a model of inescapable stress, in which microglial activation was not related to increased hypothalamic IL-6 levels [40]; and (c) nicotine exposure during lactation, which promotes paraventricular hypothalamic microglial activation related to obesity in adulthood, but with no alteration in hypothalamic IL-6 expression [41].

Lastly, two studies showed microglial suppression with decreased IL-6 expression. The first one demonstrated that the stress-induced increase in hypothalamic IL-6 and microglia activation were suppressed following benzodiazepine treatment, ameliorating anxiety, and social avoidance behavior in adult mice [42]. In the same way, exercise can ameliorate hypertension in a murine model, with a marked decrease in IL-6 expression, followed by a reduction in microglial activation in the hypothalamus [43].

Some risks of bias were strongly present in most of the articles analysed, as unclear or not founded information: (a) risk of selection bias, as randomized allocation of animals and cages; (b) risk of performance bias, as blinding of manipulators about interventions performed; (c) risk of detection bias, as random selection of animals to assess outcomes ([Supplementary-material supplementary-material-1]).

## 4. Discussion

In this review, we present a summary of data extracted from studies that evaluated IL-6 in hypothalamic microglia. The models employed in the 13 studies included in this review were divided into those considered “injury-like” stimuli, which can interfere with the hypothalamic–pituitary–adrenal (HPA) axis [32, 33, 38, 42], and others leading to phenotypic manifestations such as an increase in weight gain under a standard diet [33, 35] or anxiety-like behavior [42].

Many signaling pathways are activated by LPS or high-fat feeding [27]. Both LPS and SFAs from the diet are recognized by TLR4 in microglial cells, increasing the production and release of several inflammatory cytokines by these cells [16]. The same happens with overfeeding during lactation in neonates, which causes long-term changes that can lead to the development of obesity [44]. In this case, when adulthood is reached, basal hypothalamic IL-6 levels remain elevated, concomitant with activated hypothalamic microglia [35]. Furthermore, neonatal overfeeding beginning early in life increases hypothalamic TLR4 expression and the number of Iba-1 (microglia/macrophage-specific protein) positive cells, followed by increased expression of IL-6 following LPS challenge [33].

In addition to TLR4, microglial receptors dependent on ATP binding are also important to trigger cytokine production. Evidence indicates that microglial IL-6 production is more strongly associated with the activation of P2Y receptors [45]. A study by Mingam et al. (2008) confirmed the specificity of P2 purinoceptors to the production of cytokines, as absence of the P2X7 receptor leads to impairment only in IL-1*β* production by activated microglia but does not interfere with IL-6 production after LPS stimulus [34].

Neuroinflammation driven by infections and other systemic inflammatory events can be modulated by several ligands and specific receptors. Mice submitted to viral or bacterial lung infection drive the hepatic release of circulating amyloids, which activate PVN microglia through binding to formyl peptide receptor 2 (Fpr2), leading to a marked increase in hypothalamic IL-6 expression and exacerbated neuroinflammation [32]. Other evidence suggests that the hypothalamic distribution of receptors with multiple ligands, such as Fpr2, can contribute to these effects [46].

Although diverse pathways are related to microglial activation related to inflammation, different conditions, such as hormonal stimuli, can also result in microglial activation and increased IL-6 expression [31, 37, 47]. As shown by Ropelle et al. (2010], physical exercise increases hypothalamic IL-6 expression, which improves insulin and leptin signaling in the hypothalamus, leading to decreased food intake in rats fed with a high-fat diet [48]. Two of the reviewed studies demonstrated that IL-6 can interfere with energy balance, inhibiting food intake or reducing body weight gain, and that hormonal signaling is related to hypothalamic IL-6 expression through different mechanisms. Amylin stimulus increases IL-6 in the ventromedial hypothalamus (VMH) through binding to the microglia. Elevated IL-6 can improve leptin signaling in neurons via the phosphorylation of STAT-3, thereby reducing body weight gain [31]. Leptin itself can drive this event, as IL-6 is produced by microglia after a leptin stimulus through activation of microglial leptin receptor isoforms [47]. Furthermore, IL-6 can interact with leptin in the parabrachial nucleus, leading to reduced food intake [24]. On the other hand, mimecan can reduce food intake independent of leptin signaling, and its action is related to microglial IL-6 release in the hypothalamus [37]. Although the increases in hypothalamic IL-6 are primarily associated with inflammatory events and responses, growing evidence suggests a relationship between IL-6 and hormones involved in energy balance, which can occur in a leptin-dependent or leptin-independent manner.

Beyond the regulation of energy balance, we found several studies that reported noninflammatory outcomes of IL-6 in the CNS. One of these studies was focused on neuroplasticity via the PI3k-AKT pathway [49], while the others assessed neuroprotection and repair events [19, 50], the maintenance and control of proliferative niches close to ventricles [51], and neurogenesis in the subventricular zone mediated by IL-6 and other cytokines of microglial origin [52].

One of the reviewed studies adopted an aerobic-training protocol in spontaneously hypertensive rats presenting with hypothalamic inflammation and found a decrease in high mobility group box-1 (HMGB1, related to the injury-induced inflammatory response) and CXCR4 signaling, which ameliorates the autonomic control of blood pressure due to a reduction in microglia activation and hypothalamic IL-6 expression [43]. Indeed, anti-inflammatory events have been found to be associated with physical exercise protocols and an increase in circulating IL-6 released by the muscle [53, 54], thus reinforcing the anti-inflammatory effect of exercise and its central outcomes.

Not every inflammatory stimulus or condition is related to increased IL-6 expression in the hypothalamus, although it is classically associated with microglial activation along with IL-1*β* and TNF*α* expression. In a model of inescapable stress, microglial activation was not found to be related to an increase in hypothalamic IL-6 levels [40]. A similar finding was reported for nicotine exposure during lactation, with no relationship between long-term obesity and increased hypothalamic IL-6 expression [41]. Finally, during the aging process, an increase in microglial IL-6 production was observed in the cerebellum, cortex, and hippocampus, but not in the hypothalamus. Increased IL-6 expression in the hypothalamus did not show an age dependence [39].

Given the complexity of homeostatic maintenance and inflammatory events, an absence of microglial activity or IL-6 production can lead to impaired phenotypes. Under basal conditions, microglia ablation leads to a variety of events, including reduced neuroblast survival within the dentate gyrus of the hippocampus [55]. In the presence of severe injury, such as brain ischemia, microglia have been described as an important producer of neurotrophic factors [56] and other proteins. In a physiological context, according to the diverse microglial hypothalamic signatures, it acts like a “sentinel,” functioning as an environmental sensor and regulator of hypothalamic metabolic control [28]. As demonstrated by Silva et al. (2018), pharmacological inhibition of microglia in the CNS combined with hypoxia leads to an increase in IL-6 expression, probably due to a different cell type, such as astrocytes, resulting in alterations in the brain autonomic nuclei responsible for cardiorespiratory control [36]. Furthermore, ablation of IL-6 (knockout IL-6 mice) is related to weight gain and disturbance in glucose homeostasis during adulthood [57]. These knockout mice have lower neuronal protection in the dentate gyrus. Conversely, elevated expression of hippocampal IL-6 was found to be related to better neuronal regeneration and a better neuroprotective effect in acute lesions [58]. Furthermore, IL-6 plays a critical role in neuronal survival during early life development and adulthood [59]. Thus, there is evidence that IL-6 can exert central and peripheral functions through modulation of metabolic events, neuroprotection, and participation in regenerative/proliferative processes.

Given the plasticity of microglia [60] and the pleiotropy of IL-6 [22], studies that evaluate both require specific and accurate approaches such as conditional knockouts. In light of this, future studies should be conducted with the purpose of clarifying the behavior of microglia, as well as the effect of IL-6, under different conditions. Understanding this relationship could lead to more specific pharmacological approaches to acute and/or chronic conditions in the future, such as the management of obesity and metabolic diseases. This refinement is important in order not to disturb the homeostasis of the hypothalamic environment, as IL-6 and microglia make a remarkable contribution under normal physiological conditions.

## 5. Conclusions

**Figure 1:**
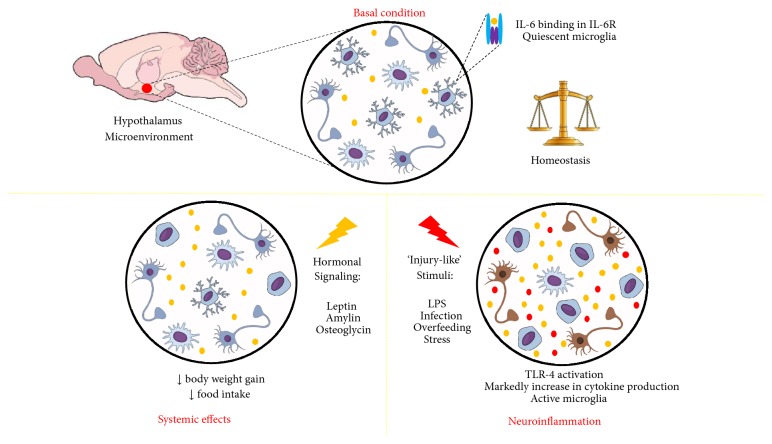
Summary of findings. IL-6 (represented by yellow dots) in hypothalamic microenvironment. In basal conditions, binding in its receptor have correlation with homeostatic maintenance of microenvironment. During hormonal signaling, temporary increases in IL-6 levels were related to systemic effects provide by hormonal signaling. During “injury-like” stimuli, neuroinflammation is characterized by marked increase in IL-6 and other cytokine production, microglial activation, and TLR-4 activation.

Advances in our understanding of microglial IL-6 hypothalamic expression and its functions are important for the interpretation of hypothalamic responses under diverse stimuli. Taken together, our review identified three main contexts where microglial activity and IL-6 expression are strongly related: (1) basal levels of hypothalamic IL-6 and microglial function are important to maintain environmental homeostasis; (2) some hormones can activate microglia and increase IL-6 expression to improve hormonal signaling in the hypothalamus; and (3) under conditions of acute or sustained inflammatory conditions, IL-6 expression and microglia activation will be increased together with other inflammatory markers such as TNF*α* and IL-1*β*, generating neuroinflammatory responses (Figure 1).

## Figures and Tables

**Table 1:** Systematic review of data search.

	Source	Subject	Models Interventions	Methods of analysis: mRNA measurement	Methods of analysis: Protein measurement	IL-6 measurement, tissue	Microglia status, tissue	Microglia IL-6 producer	Outcome
IL-6 increased and microglia activated	Wang, H. 2018	Viral-bacterial lung co-infection	mice, adults/ primary microglial cell culture *Influenza A, Streptococcus. pneumoniae*, SAA exposure	qPCR - Hypothalamus and primary cell culture: *Tnf, Il1b, Il6, Ccl2, Fpr2*, S*aa1*	IHC - PVN: IBA1, GFAP, TNF-*α*, SAA	mRNA expression increased, hypothalamus (P < 0.05)	Activated, PVN (P < 0.05)	Yes (P < 0.05)	Neuroinflammation
	Le Foll, C. 2015	Amylin exposure	rats and mice, adults/ VMH explants/ VMH astrocytes/ VMN neurons/ cortical and hypothalamic microglia Amylin exposure	qPCR - ARC, VMH, VMN, cell culture:: *Il-1b, Il-6, Il-10, Tnf, Lif, Cntf, Gp130, Ctr1a, Ctr1b, Ramp1, Ramp2, Ramp3, Lepr-b, Socs3, Ins-r, Npy, Agrp, Pomc.*	ICC - ARC, VMN: pSTAT3 Immunoassay - cell culture medium: IL-1*β*, IL-6, IL-10, TNF-*α*	mRNA expression increased, VMN (P < 0.05)	Activated, Cortical ( P < 0.05)	Yes	Decreased body weight gain
	Ziko, I. 2014	LPS exposure in adults Neonatally overfed	rats, pups and adults Neonatal overfeeding model LPS exposure	qPCR - hypothalamus: *Tlr4, Nfkb,Il1b, Il1ra, Il6, Tnf*	IHC - DMH, LH, VMH: IBA1	mRNA expression increased, hypothalamus (P < 0.05)	Activated, PVN (P < 0.05)	No data	Increased body weight in small litters and adults
	Tapia-González, S. 2011	Neonatal overfeeding	rats, pups Neonatal overfeeding model		WB - hypothalamus: IL-6, p-IkB IHC - ARC, ME, VMH: MHC-II	Protein expression increased, hypothalamus (P < 0.01)	Activated, hypothalamus, (P<0,05)	No data	Overweight
	Mingam, R. 2008	LPS exposure in P2X7R knockout model	mice, adult/ primary glial cell culture LPS exposure	qPCR - hypothalamus:* Il-6, Il-1b, Tnf*	ELISA - cell culture medium: IL-1*β* IHC - cell culture: CD11b, CD68, IL-1*β*, GFAP, WB - cell culture medium: IL-1*β*	Protein and mRNA expression increased, whole brain (P < 0.05)	Activated (P < 0.05)	Yes (P < 0.05)	Decreased IL-1*β* release

IL-6 increased without microglia information	Cao, H. M. 2015	Mimecan exposure	Rats and mice, adults primary neurons cell culture N9 cells Mimecan exposure	qPCR - hypothalamus: *Il1-b, Il-6* qPCR - primary cell culture: *Il1-b, Il-6, Socs3* qPCR qPCR -N9 cell culture: *Il1-b, Il-6, Socs3*	WB - hypothalamus: SOCS3	mRNA expression increased, hypothalamus (P < 0.05 / P < 0.01)	No data	Yes (P < 0.01)	Anorexia
	Roque, A. 2015	Early life stress	rats, pups Maternal separation model	qPCR - hypothalamus: *Il1-b, Il-6, Tnf*	-	mRNA expression increased, hypothalamus (P < 0.01)	No data	No data	Stress can dysregulate the HPA axis

IL-6 increase and microglia suppressed	Silva, T.M. 2017	Minocycline in acute hypoxia	rats, adults Pretreatment with Minocycline followed by exposition to acute hypoxia	qPCR - mice PVH: *Il-1b, Il-6, Tnf, Mmp9, Cd3, Hprt*	IHC: TH, c-Fos	mRNA expression increased, PVH (P < 0.05)	Suppressed (P < 0.05)	No	Disrupted organization of breath activity

IL-6 unaltered and microglia activated	Younes-Rapozo, V. 2015	Long-term effects of nicotine exposure during lactation in offspring	rats, pups Nicotine in dams.	-	IF - ARC, PVN, LH, PE: GFAP, IBA-1, CX3CR1, MCP-1, IL-6	Protein expression had no change, ARC, PE, PVN, LH	Activated, in PVN (P < 0.05)	No data	Obesity
	Sugama, S. 2007	Inescapable stress	rats and mice, adults restraint combined with water immersion stress	qPCR - hypothalamus: *Il-1b, Il-6, Inos*	IF: IL1-*β*, IL-6, iNOS IHC: OX42, CD11b	mRNA expression had no change, hypothalamus	Activated (P < 0.001)	No	Hypothalamic microglia activation
	Ye, S. M. 1999	Aging	mice, juvenile, adult, and aged/ primary cell culture of whole brains Basal conditions	RT-PCR - glial cell culture: *Il-6*	ELISA glial cell culture medium: IL-6 IHC: GFAP, MAC-1(CD11B) Flow Cytometry glial cell culture: GFAP, MAC-1 ELISA tissue homogenates: IL-6 Hybridoma bioassay: IL-6	Protein expression had no change in the hypothalamus	Activated, all brain (P < 0.05)	Yes (P < 0.01)	chronic inflammation in whole brain without changes in the hypothalamus

IL-6 decreased and microglia suppressed	Ramirez, K. 2016	Benzodiazepines treatment in Repeated social stress condition	Mice, adult Exposed to stress condition and treated with benzodiazepines	qPCR - hypothalamus: *Il1-b, Il-6, Tnf*	-	mRNA expression decreased, hypothalamus (P < 0.05)	Suppressed, whole brain (P < 0.05)	Yes (P < 0.05)	reversal of stress-induced behavioral
	Santos Masson, G. 2015	Short-term exercise training in arterial hypertension model	Rat, adult 2 weeks of exercise model in spontaneously hypertensive rats	-	WB - PVN: CXCR4, ERK1/ 2 pERK1/ 2, HMGB1, IL-6, p-IKBa, SDF-1, TNF-*α* IF: HMGB1, IBA-1	Protein expression decreased, PVN (P < 0.01)	Suppressed, PVN (P < 0.01)	No data	Control of arterial hypertension

Legend: ARC: arcuate nucleus, LPS: lipopolysaccharide, HPA: hypothalamic–pituitary–adrenal ME: median eminence, PVN: paraventricular nucleus, SAA: serum amyloid A, VMH: ventromedial hypothalamus, VMN: ventromedial nucleus.

## Supplementary Materials

Supplementary Materials Figure S1: PRISMA flow diagram. Table S1: Excluded and included articles. Table S2: Evaluation of Risk of Bias, using SYRCLE's risk of bias tool for animal studies. Table S3: PRISMA Checklist.
