# Supplementary material for: Interleukin-6 Expression by Hypothalamic Microglia in Multiple Inflammatory Contexts: A Systematic Review
Source: Biomed Res Int. 2019 Aug 22;2019:1365210. doi: 10.1155/2019/1365210 (PMC6724433; doi:10.1155/2019/1365210)
Supplement: Supplementary Materials — Figure S1: PRISMA flow diagram. Table S1: Excluded and included articles. Table S2: Evaluation of Risk of Bias, using SYRCLE's risk of bias tool for animal studies. Table S3: PRISMA Checklist. [file 1365210.f1.docx]

**Supplementary information**

Information 1: Web address of the findings.

PubMed search.

https://www.ncbi.nlm.nih.gov/PubMed?term=((hypothalamus%5BMeSH%20Terms%5D)%20AND%20interleukin-6%5BMeSH%20Terms%5D)%20AND%20microglia%5BMeSH%20Terms%5D

Web Of Science search.

https://apps.webofknowledge.com/Search.do?product=WOS&SID=5DnpQOqknBU5JnYtpg9&search_mode=GeneralSearch&prID=e140641e-0f8a-4638-b18b-442de5561349


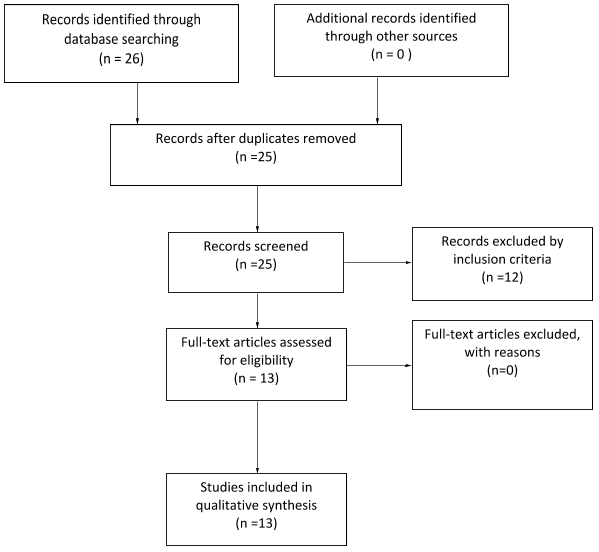


Figure S1: PRISMA flow diagram.

*(Based on: Moher D, Liberati A, Tetzlaff J, Altman DG, The PRISMA Group (2009). Preferred Reporting Items for Systematic Reviews and Meta-Analyses: The PRISMA Statement. PLoS Med 6(7): e1000097. doi:10.1371/journal.pmed1000097)*

Table S1: Excluded and included articles.

|  | **1st author** | **Year** | **Article Title** | **Accepted/Rejected** |
| --- | --- | --- | --- | --- |
| 1 | Tapia-González S | 2011 | Activation of microglia in specific hypothalamic nuclei and the cerebellum of adult rats exposed to neonatal overnutrition. | accepted |
| 2 | Santos Masson G | 2015 | Aerobic training normalizes autonomic dysfunction, HMGB1 content, microglia activation and inflammation in hypothalamic paraventricular nucleus of SHR. | accepted |
| 3 | Le Foll C | 2014 | Amylin-induced central IL-6 production enhances ventromedial hypothalamic leptin signaling. | accepted |
| 4 | Soares D | 2013 | Chemokine ligand (CCL)-3 promotes an integrated febrile response when injected within pre-optic area (POA) of rats and induces calcium signaling in cells of POA microcultures but not TNF-α or IL-6 synthesis. | Rejected, excluded by inclusion criteria: Didn't explore either hypothalamus or IL-6 |
| 5 | Schöning B | 1999 | Continuous infusion of proinflammatory cytokines into the brain to study brain cytokine induced local and systemic immune effects. | Rejected, excluded by inclusion criteria: Didn't explore IL-6 |
| 6 | Levkovitz Y | 2014 | Early post-stressor intervention with minocycline, a second-generation tetracycline, attenuates post-traumatic stress response in an animal model of PTSD | Rejected, excluded by inclusion criteria: Didn't explore microglia |
| 7 | Bryan KJ | 2008 | Expression of CD74 is increased in neurofibrillary tangles in Alzheimer's disease | Rejected, excluded by inclusion criteria: Didn't explore either hypothalamus or IL-6 |
| 8 | Myers R | 1994 | FEVER AND FEEDING IN THE RAT - ACTIONS OF INTRAHYPOTHALAMIC INTERLEUKIN-6 COMPARED TO MACROPHAGE INFLAMMATORY PROTEIN-1-BETA (MIP-1-BETA) | Rejected, excluded by inclusion criteria: Didn't explore microglia |
| 9 | Ramirez K | 2015 | GABAergic modulation with classical benzodiazepines prevent stress-induced neuro-immune dysregulation and behavioral alterations. | accepted |
| 10 | Holguin A | 2004 | HIV-1 gp120 stimulates proinflammatory cytokine-mediated pain facilitation via activation of nitric oxide synthase-1 (nNOS) | Rejected, excluded by inclusion criteria: Didn't explore either hypothalamus or microglia |
| 11 | Navarra P | 2006 | Hydroxyurea induces vasopressin release and cytokine gene expression in the rat hypothalamus | Rejected, excluded by inclusion criteria: Didn't explore either microglia or IL-6 |
| 12 | Mingam R | 2008 | In vitro and in vivo evidence for a role of the P2X7 receptor in the release of IL-1 beta in the murine brain. | accepted |
| 13 | Wang H | 2018 | Increased hypothalamic microglial activation after viral-induced pneumococcal lung infection is associated with excess serum amyloid A production | accepted |
| 14 | Ye SM | 1999 | Increased interleukin-6 expression by microglia from brain of aged mice | accepted |
| 15 | Carrasco, J | 1998 | Localization of metallothionein-I and -III expression in the CNS of transgenic mice with astrocyte-targeted expression of interleukin 6 | Rejected, excluded by inclusion criteria: Didn't explore hypothalamus |
| 16 | Roque A | 2015 | Maternal separation activates microglial cells and induces an inflammatory response in the hippocampus of male rat pups, independently of hypothalamic and peripheral cytokine levels. | accepted |
| 17 | Cao HM | 2015 | Mimecan, a Hormone Abundantly Expressed in Adipose Tissue, Reduced Food Intake Independently of Leptin Signaling. | accepted |
| 18 | Silva TM | 2017 | Minocycline alters expression of inflammatory markers in autonomic brain areas and ventilatory responses induced by acute hypoxia | accepted |
| 19 | Xiong X | 2013 | Mitigation of Murine Focal Cerebral Ischemia by the Hypocretin/Orexin System is Associated With Reduced Inflammation | Rejected, excluded by inclusion criteria: Didn't explore hypothalamus |
| 20 | Younes-Rapozo V | 2015 | Neonatal Nicotine Exposure Leads to Hypothalamic Gliosis in Adult Overweight Rats | accepted |
| 21 | Ziko I | 2014 | Neonatal overfeeding alters hypothalamic microglial profiles and central responses to immune challenge long-term | accepted |
| 22 | Ott D | 2010 | Neurons and glial cells of the rat organum vasculosum laminae terminalis directly respond to lipopolysaccharide and pyrogenic cytokines. | Rejected, excluded by inclusion criteria: Didn't explore hypothalamus |
| 23 | Duffy CM | 2015 | Role of orexin A signaling in dietary palmitic acid-activated microglial cells. | Rejected, excluded by inclusion criteria: Didn't explore hypothalamus |
| 24 | Ott D | 2012 | The viral mimetic polyinosinic:polycytidylic acid (poly I:C) induces cellular responses in primary cultures from rat brain sites with an incomplete blood-brain barrier. | Rejected, excluded by inclusion criteria: Didn't explore hypothalamus |
| 25 | Sugama S | 2007 | Stress induced morphological microglial activation in the rodent brain: Involvement of interleukin-18 | accepted |

Table S2: Evaluation of Risk of Bias, using SYRCLE’s risk of bias tool for animal studies.

| Type of Bias | Questions | Cao HM, 2015 | Le Foll C, 2014 | Mingam R, 2008 | Ramirez K, 2015 | Roque A, 2015 | Santos Masson G, 2015 | Silva TM, 2017 | Sugama S, 2007 | Tapia Gonzalez S, 2011 | Wang H, 2018 | Ye SM, 1999 | Younes-Rapozo V, 2015 | Ziko I, 2014 |
| --- | --- | --- | --- | --- | --- | --- | --- | --- | --- | --- | --- | --- | --- | --- |
| Selection Bias | Was the allocation sequence adequately generated and applied? | u | u | u | u | u | u | u | u | u | u | u | u | y |
|  | Were the groups similar at baseline or were they adjusted for confounders in the analysis? | y | y | y | y | y | y | y | y | y | y | y | y | y |
|  | Was the allocation to the different groups adequately concealed during? | n | n | n | n | n | n | n | n | n | n | n | n | n |
| Performance Bias | Were the animals randomly housed during the experiment? | u | u | u | u | u | u | u | u | u | u | u | u | u |
|  | Were the caregivers and/or investigators blinded from knowledge which intervention each animal received during the experiment? | n | n | n | n | n | n | n | n | n | n | n | n | n |
| Detection Bias | Were animals selected at random for outcome assessment? | n | n | n | n | n | n | n | n | n | n | n | n | n |
|  | Was the outcome assessor blinded? (image counting experiments) | - | y | n | - | u | u | n | u | n | y | n | y | y |
| Attrition Bias | Were incomplete outcome data adequately addressed? | y | y | y | y | y | u | y | u | y | y | y | y | y |
| Reporting Bias | Are reports of the study free of selective outcome reporting? | y | y | y | y | y | u | y | y | y | y | u | y | y |
| Other | Was the study apparently free of other problems that could result in high risk of bias? | y | y | y | y | y | y | y | y | y | y | y | y | y |
|  | Legend: n=no (high risk of bias); y=yes (low risk of bias); u=unclear (unclear risk of bias). |  |  |  |  |  |  |  |  |  |  |  |  |  |

Based on Hooijmans et al (2014). SYRCLE’s risk of bias tool for animal studies. BMC Medical Research Methodology 2014, 14:43.available at: <http://www.biomedcentral.com/1471-2288/14/43>

Table S3: PRISMA Checklist

| **Section/topic** | **#** | **Checklist item** | **Reported on page #** |
| --- | --- | --- | --- |
| **TITLE** | | |  |
| Title | 1 | Identify the report as a systematic review, meta-analysis, or both. | 1 |
| **ABSTRACT** | | |  |
| Structured summary | 2 | Provide a structured summary including, as applicable: background; objectives; data sources; study eligibility criteria, participants, and interventions; study appraisal and synthesis methods; results; limitations; conclusions and implications of key findings; systematic review registration number. | 2 |
| **INTRODUCTION** | | |  |
| Rationale | 3 | Describe the rationale for the review in the context of what is already known. | 3-4 |
| Objectives | 4 | Provide an explicit statement of questions being addressed with reference to participants, interventions, comparisons, outcomes, and study design (PICOS). | 4 |
| **METHODS** | | |  |
| Protocol and registration | 5 | Indicate if a review protocol exists, if and where it can be accessed (e.g., Web address), and, if available, provide registration information including registration number. | - |
| Eligibility criteria | 6 | Specify study characteristics (e.g., PICOS, length of follow-up) and report characteristics (e.g., years considered, language, publication status) used as criteria for eligibility, giving rationale. | 5 |
| Information sources | 7 | Describe all information sources (e.g., databases with dates of coverage, contact with study authors to identify additional studies) in the search and date last searched. | 5 |
| Search | 8 | Present full electronic search strategy for at least one database, including any limits used, such that it could be repeated. | 5 |
| Study selection | 9 | State the process for selecting studies (i.e., screening, eligibility, included in systematic review, and, if applicable, included in the meta-analysis). | 5 |
| Data collection process | 10 | Describe method of data extraction from reports (e.g., piloted forms, independently, in duplicate) and any processes for obtaining and confirming data from investigators. | 5 |
| Data items | 11 | List and define all variables for which data were sought (e.g., PICOS, funding sources) and any assumptions and simplifications made. | 5 |
| Risk of bias in individual studies | 12 | Describe methods used for assessing risk of bias of individual studies (including specification of whether this was done at the study or outcome level), and how this information is to be used in any data synthesis. | 5 |
| Summary measures | 13 | State the principal summary measures (e.g., risk ratio, difference in means). | Doesn’t apply |
| Synthesis of results | 14 | Describe the methods of handling data and combining results of studies, if done, including measures of consistency (e.g., I^2^) for each meta-analysis. | Doesn’t apply |

Page 1 of 2

| **Section/topic** | **#** | **Checklist item** | **Reported on page #** |
| --- | --- | --- | --- |
| Risk of bias across studies | 15 | Specify any assessment of risk of bias that may affect the cumulative evidence (e.g., publication bias, selective reporting within studies). | - |
| Additional analyses | 16 | Describe methods of additional analyses (e.g., sensitivity or subgroup analyses, meta-regression), if done, indicating which were pre-specified. | Doesn’t apply |
| **RESULTS** | | |  |
| Study selection | 17 | Give numbers of studies screened, assessed for eligibility, and included in the review, with reasons for exclusions at each stage, ideally with a flow diagram. | Supplementary material |
| Study characteristics | 18 | For each study, present characteristics for which data were extracted (e.g., study size, PICOS, follow-up period) and provide the citations. | 6 |
| Risk of bias within studies | 19 | Present data on risk of bias of each study and, if available, any outcome level assessment (see item 12). | - |
| Results of individual studies | 20 | For all outcomes considered (benefits or harms), present, for each study: (a) simple summary data for each intervention group (b) effect estimates and confidence intervals, ideally with a forest plot. | 6-7 |
| Synthesis of results | 21 | Present results of each meta-analysis done, including confidence intervals and measures of consistency. | Doesn’t apply |
| Risk of bias across studies | 22 | Present results of any assessment of risk of bias across studies (see Item 15). | - |
| Additional analysis | 23 | Give results of additional analyses, if done (e.g., sensitivity or subgroup analyses, meta-regression [see Item 16]). | Doesn’t apply |
| **DISCUSSION** | | |  |
| Summary of evidence | 24 | Summarize the main findings including the strength of evidence for each main outcome; consider their relevance to key groups (e.g., healthcare providers, users, and policy makers). | 10 |
| Limitations | 25 | Discuss limitations at study and outcome level (e.g., risk of bias), and at review-level (e.g., incomplete retrieval of identified research, reporting bias). | - |
| Conclusions | 26 | Provide a general interpretation of the results in the context of other evidence, and implications for future research. | 12 |
| **FUNDING** | | |  |
| Funding | 27 | Describe sources of funding for the systematic review and other support (e.g., supply of data); role of funders for the systematic review. | 13 |
